# Clinical evaluation of corneal elasticity and true IOP using a dual-applanation tonometer

**DOI:** 10.3389/fopht.2026.1811004

**Published:** 2026-05-08

**Authors:** Ciro Caruso, Gaetano Barbaro, Mario Troisi, Francesco Matarazzo, Ciro Costagliola

**Affiliations:** 1 Clinica Oculistica, Pellegrini Hospital, ASL Naples 1, Naples, Italy; 2 Unità di Oculistica, Istitute of Refractive and Ophthalmologic Surgery Naples, Naples, Italy; 3 Unità di Oculistica (UOC), Ruggi d’Aragona Hospital, Salerno, Italy; 4 Dipartimento di Fisica “Ettore Pacini”, Università degli Studi di Napoli Federico II, Naples, Italy; 5 Dipartimento di Neuroscienze, Scienze Riproduttive ed Odontostomatologiche, Università degli Studi di Napoli Federico II, Naples, Italy

**Keywords:** corneal biomechanics, dual applanation, glaucoma, Goldmann tonometry, intraocular pressure, refractive surgery, Young’s modulus

## Abstract

**Purpose:**

To evaluate the clinical feasibility of a modified Goldmann applanation system capable of estimating true intraocular pressure and corneal elasticity *in vivo*.

**Methods:**

A dual-applanation prism was developed, allowing two sequential IOP readings under identical optical conditions. A mathematical model was applied to compute true intraocular pressure and the corneal Young’s modulus measured applanation forces, corneal thickness and curvature. Fifty-three healthy eyes were examined. Model-derived true intraocular pressure values were compared with standard GAT, Pascal dynamic contour tonometry and corneal Young’s modulus.

**Results:**

Mean IOPT was 15.72 ± 2.44 mmHg, significantly higher than GAT measurements (14.86 ± 2.71 mmHg; mean difference +0.86 ± 1.82 mmHg, p = 0.0012). IOPT showed strong agreement with Pascal DCT (15.60 ± 2.35 mmHg), with minimal bias (+0.15 ± 1.63 mmHg, p = 0.499) and high correlation (r = 0.93, p < 0.0001). Measurement variance was lower for IOPT than for GAT, indicating improved precision. The mean *in vivo* corneal Young’s modulus was 0.18 ± 0.04 MPa (range 0.057 MPa to 0.296 MPa.), consistent with physiological values. Young’s modulus showed significant age-related variation (p < 0.001) and a moderate inverse correlation with IOPT (r = −0.53, p < 0.001), while no significant sex-related differences were observed.

**Conclusion:**

This dual-applanation system enables real-time *in vivo* estimation of both true intraocular pressure and corneal elasticity. The model provides a physically grounded correction to Goldmann tonometry and introduces a new clinically accessible method to assess corneal biomechanics.

## Highlights

- A novel dual-zone applanation prism enables sequential IOP measurements.
- The model computes both true intraocular pressure (IOPT) and corneal Young’s modulus (E).
- IOPT shows strong agreement with Pascal tonometry, reducing GAT bias.
- *In vivo* biomechanical estimation is feasible with standard slit-lamp tonometry.
- The system provides clinically relevant corneal elasticity data for personalized care.

## Introduction

The Goldmann applanation tonometer (GAT) remains the gold standard for IOP measurement in clinical practice due to its simplicity and repeatability. However, its accuracy is compromised by corneal properties, including central corneal thickness (CCT), curvature, and biomechanical behavior, which introduce systematic errors and inter-individual variability in the estimation of true intraocular pressure (1, 2).

To address these limitations, several procedures and correction strategies have been proposed. Dynamic contour tonometry (DCT), exemplified by the Pascal device, measures IOP by matching the contour of the probe tip to the cornea and is considered less sensitive to CCT variations (3). The Ocular Response Analyzer (ORA) evaluates corneal hysteresis and resistance factor based on bidirectional air puff deformation, providing biomechanically adjusted IOP estimates (4). The Corvis ST employs high-speed Scheimpflug imaging to record corneal deformation dynamics, from which biomechanical parameters such as stiffness, deformation amplitude, and stress–strain index can be inferred (5).

Although several instruments have been developed to provide additional information on corneal biomechanics, their widespread use in routine clinical practice remains limited due to factors such as higher cost, increased technological complexity, and reduced accessibility compared with conventional applanation tonometry. Goldmann applanation tonometry (GAT) remains the clinical gold standard for measuring intraocular pressure (IOP), although its accuracy can be influenced by corneal biomechanical properties and thickness. In an attempt to reduce these biomechanical influences, Dynamic Contour Tonometry (DCT) was developed as an alternative method designed to measure IOP with reduced dependence on corneal properties. Moreover, many of the indices they provide are not direct physical quantities, but rather composite metrics derived from proprietary algorithms, limiting their interpretability and reproducibility across platforms (6). Ko and colleagues proposed an indentation-based approach to estimate an intraocular-pressure–dependent corneal “tangent modulus” *in vivo* (7).

McCafferty et al. introduced the Correcting Applanation Tonometry Surface (CATS) prism, a geometrically optimized version of the GAT prism that reduces IOP measurement error in eyes with low CCT or low corneal resistance factor (CRF) (8).

The aim of this study is to evaluate the clinical feasibility and accuracy of a dual-applanation system in estimating IOPT and corneal elasticity *in vivo*. Specifically, we compare model-derived IOPT values to those obtained from GAT and Pascal DCT, and assess the reproducibility and physiological plausibility of the derived corneal Young’s modulus (E) in a cohort of healthy subjects.

## Materials and methods

### Mathematical rationale and model derivation

A physical–mathematical model has been developed to simulate the forces acting on the cornea during applanation (9–11). The model includes four principal forces:

1. the IOP acting on the posterior corneal surface.
2. the elastic resistance of the corneal tissue.
3. the adhesive force from the tear film surface tension.
4. the applanating force required to flatten a standardized corneal area.

By solving this model using biometric inputs and two independent applanation readings, it is possible to simultaneously estimate IOPT and the *in vivo* corneal Young’s modulus (E), a parameter with strong clinical relevance in glaucoma risk profiling, refractive surgery screening, and keratoconus monitoring (12).

**Figure 1:**
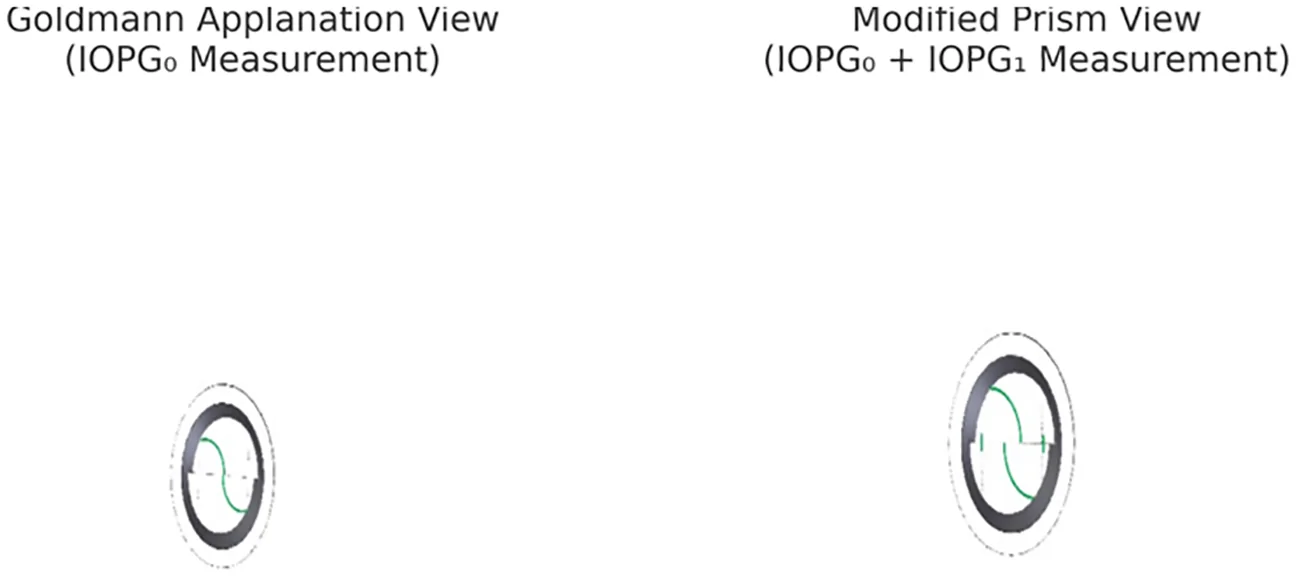
Visual appearance through the Goldmann tonometer prism (left) and through the modified dual-measurement prism (right). Left image IOPG_0_: Standard applanation interface with a 3.06 mm diameter contact zone, showing the typical alignment of two semicircular fluorescein rings used to determine IOPG_0_. Right image IOPG_1_: Appearance through the newly developed prism, which retains the standard central measurement (IOPG_1_) while simultaneously revealing two additional peripheral arcs corresponding to an outer concentric applanated zone of 3.60 mm diameter. This configuration enables a second sequential pressure measurement (IOPG_1_). This dual-applanation system enables acquisition of two pressure values during a single applanation session.

**Figure 2:**
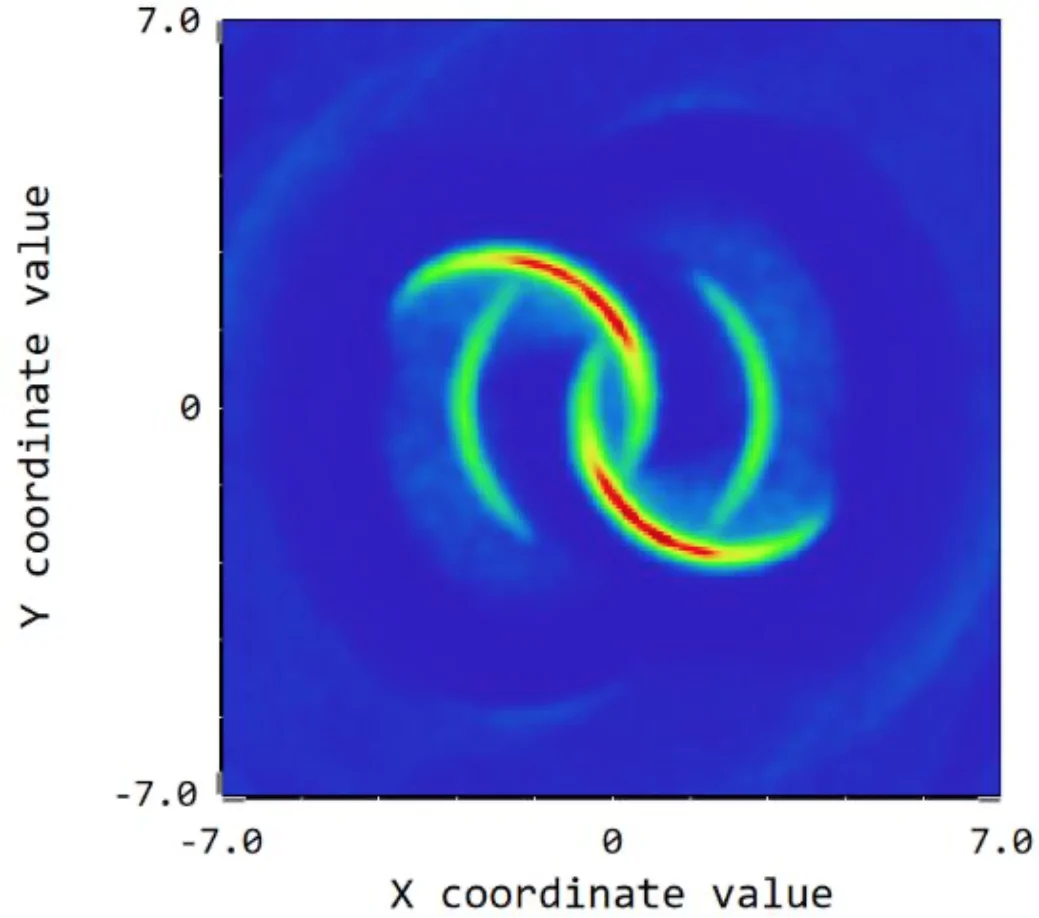
Each applanation area corresponds to a specific contact diameter on the cornea, with both images observed simultaneously through a slit-lamp interface. This configuration generates two distinct fluorescein patterns (semicircles=IOPG_0_ and arcs=IOPG_1_).

To acquire the necessary dual applanation values, a modified optical prism was engineered. A dual-zone prism allows for two concentric corneal applanations under the same slit-lamp observation: an inner 3.06 mm zone corresponding to standard GAT, and an outer 3.60 mm zone. The result is a dual fluorescein image—semicircles and arcs—captured within a single maneuver (**Figures 1**, **2**). The two corresponding pressures (IOPG_0_ and IOPG_1_) serve as input for the model, enabling real-time biomechanical analysis without altering routine clinical workflow (13).

This study is based on a physical–mathematical model that describes the applanation mechanics involved in Goldmann tonometry. When the cornea is flattened by the tonometer prism, four primary forces act upon the system:

1. The intraocular pressure force (F_1_), which corresponds to the internal pressure of the aqueous humor acting on the applanated area:


$${\text{F}}_{1}\;=\text{ IOP }\times \text{ π }\times {\text{g}}^{2}$$

2. The elastic reaction force (F_2_), generated by the corneal deformation during applanation, derived from spherical shell elasticity theory:


$${\text{F}}_{2}\;=\;(\text{E }\times \text{ h }\times {\text{g}}^{4})\;/\text{ a}$$

where E is the Young’s modulus of the cornea, h is the central corneal thickness, g is the radius of the applanated zone, and a is the mean apical curvature radius.

3. The tear film adhesion force (F_3_), which accounts for the capillary force produced by the tear meniscus at the applanation edge, estimated by Laplace’s law for surface tension:


$${\text{F}}_{3}\;=\;2\;\times \text{ π }\times \text{ g }\times \text{ λ }\times \text{ cos}(\text{θ})$$

where λ is the tear film surface tension (typically 0.044–0.050 N/m), and θ ≈ 0 assuming full wettability.

4. The external applanation force (F_4_), which is the force applied by the tonometer and balances the other forces:


$${\text{F}}_{4}={\text{F}}_{1}+{\text{F}}_{2}-{\text{F}}_{3}$$

From these relationships, the IOPT and the E measured by the dual applanation prism can be modeled as Equation 1:

1 $$IOPT≅IOPG_{0}+\frac{IOPG_{0}\cdot g_{12}-IOPG_{1}\cdot g_{02}}{\frac{g_{14}}{g_{02}}-g_{12}}+4.0mmHg\cdot \frac{g_{12}+g_{02}}{g_{12}}$$

Conversely, the Young’s modulus of the cornea can be estimated from measured pressures and corneal geometry, as Equation 2:

2 $$E≅\frac{a^{3}}{h}\cdot \frac{IOPG_{1}\cdot (1-\frac{g_{02}}{g_{12}})-IOPG_{0}\cdot (\frac{g_{12}}{g_{02}}+1)+4.0mmHg\cdot \frac{g_{12}+g_{02}}{g_{12}}\cdot (\frac{g_{12}}{g_{02}}-1)}{\frac{g_{14}}{g_{02}}-g_{02}}$$

g is the radius of the applanated circular area, defined as g_0_ = 1.53 mm, which corresponds to an applanation diameter of 3.06 mm and represents the IOP measured with the standard Goldmann prism. g_1_ = 1.8 mm corresponds to an applanation diameter of 3.6 mm and represents the IOP measured sequentially using the dual-applanation prism design. h is the central corneal thickness, and a is the mean apical curvature radius. This analytical model shows that the error introduced by GAT increases with the product E × h and inversely with the cube of the curvature radius a.

### Modified device design and experimental setup

A modified Goldmann tonometer prism was developed, featuring two concentric applanation zones to allow for sequential measurements under identical condition. A schematic diagram illustrating the dual-applanation system, including the central and peripheral contact zones, has been added to clarify the geometric modifications described in this section.

All tonometric measurements were performed by a single experienced glaucoma specialist to minimize inter-operator variability, which is known to influence applanation-based intraocular pressure measurements.

- IOPG_0_ = First applanation (g_0_ = 1.53 mm): standard GAT measurement yielding IOPG_0_ - IOPG_1_ = Second applanation (g_1_ = 1.80 mm): expanded zone yielding IOPG_1_. IOPG_1_ is the applanation pressure measured at the peripheral contact zone, using the outer diameter of the dual-zone prism.
- IOPT = it is not a direct measurement but a calculated value derived from the ratio of IOPG_1_ to IOPG_0_ using the analytical model described in Eq. 1.

Both IOPG_0_ and IOPG_1_ values were obtained sequentially during a single measurement session using the custom-designed prism, both images observed through a slit-lamp interface.

The study included 53 eyes from healthy volunteers. All measurements were performed under standardized lighting and fixation conditions, using the modified prism installed on a standard slit-lamp tonometer. Corneal topography and pachymetry were obtained prior to applanation, and Pascal dynamic contour tonometry was used for comparison.

### Clinical population and data analysis prospective, nonrandomized study comparing IOP measurements from a dual-applanation prism and Pascal tonometer was conducted

The study adhered to the tenets of the Declaration of Helsinki. Written informed consent was obtained from all participants prior to inclusion in the study. The procedures consisted exclusively of non-invasive ophthalmic examinations routinely used in clinical practice. According to local regulations governing observational studies involving standard diagnostic procedures in healthy volunteers, formal approval by an institutional ethics committee was not required. A total of 53 eyes from healthy volunteers were examined. Inclusion criteria were: normal corneal transparency, best corrected visual acuity ≥ 8/10, absence of ocular surface disease, and equal IOP in both eyes. This corresponded to a difference of ≤1.0 mmHg as measured by Goldmann Applanation Tonometry (GAT). In cases where both eyes met this criterion, the right eye was selected by default for analysis. Only subjects with a central corneal thickness (CCT) ≥ 500 μm, as measured by ultrasound pachymetry, were included in the study to ensure adequate corneal structural integrity for applanation-based measurements. Only patients whose average corneal curvature calculated as the arithmetic mean between the anterior and posterior corneal radii—fell within the range of 7.10 to 7.15 mm were included.

Exclusion criteria included: previous ocular surgery, corneal scarring, keratoconus, ocular hypertension > 30 mmHg, unstable tear film, or current antiglaucomatous treatment. Demographic and baseline parameters included sex (F/M), age group (20–39 years or ≥ 40 years), IOPG_0_ (Goldmann), IOPG_1_ (modified cone) and CCT. Demographic and baseline parameters of the study population are summarized in **Table 1**.

The Pascal Dynamic Contour Tonometer (DCT) was selected as the comparator for IOPT because it is considered one of the most reliable device for estimating true intraocular pressure, as it minimizes the influence of corneal properties such as thickness and rigidity. Unlike GAT, the Pascal device measures IOP through a contour-matched transducer, providing a reference that is less dependent on corneal biomechanics and therefore suitable for validating biomechanically corrected IOP measurements like IOPT.

### Statistical analysis

Statistical analyses were performed using MedCalc Statistical Software version 19.2.6 (MedCalc Software Ltd, Ostend, Belgium). All values are presented as mean ± standard deviation (SD), unless otherwise stated. Normality of data distribution was assessed using the Shapiro-Wilk test. Paired t-tests were used to compare intraocular pressure values obtained by different tonometric methods. Pearson correlation coefficients (r) were calculated to evaluate linear relationships between variables. Agreement between methods was assessed using Bland–Altman plots, and variance comparisons were performed using F-tests. A p-value less than 0.05 was considered statistically significant.

## Results

Comparison between IOPT and Goldmann applanation tonometry (IOPG_0_).

**Figure 3:**
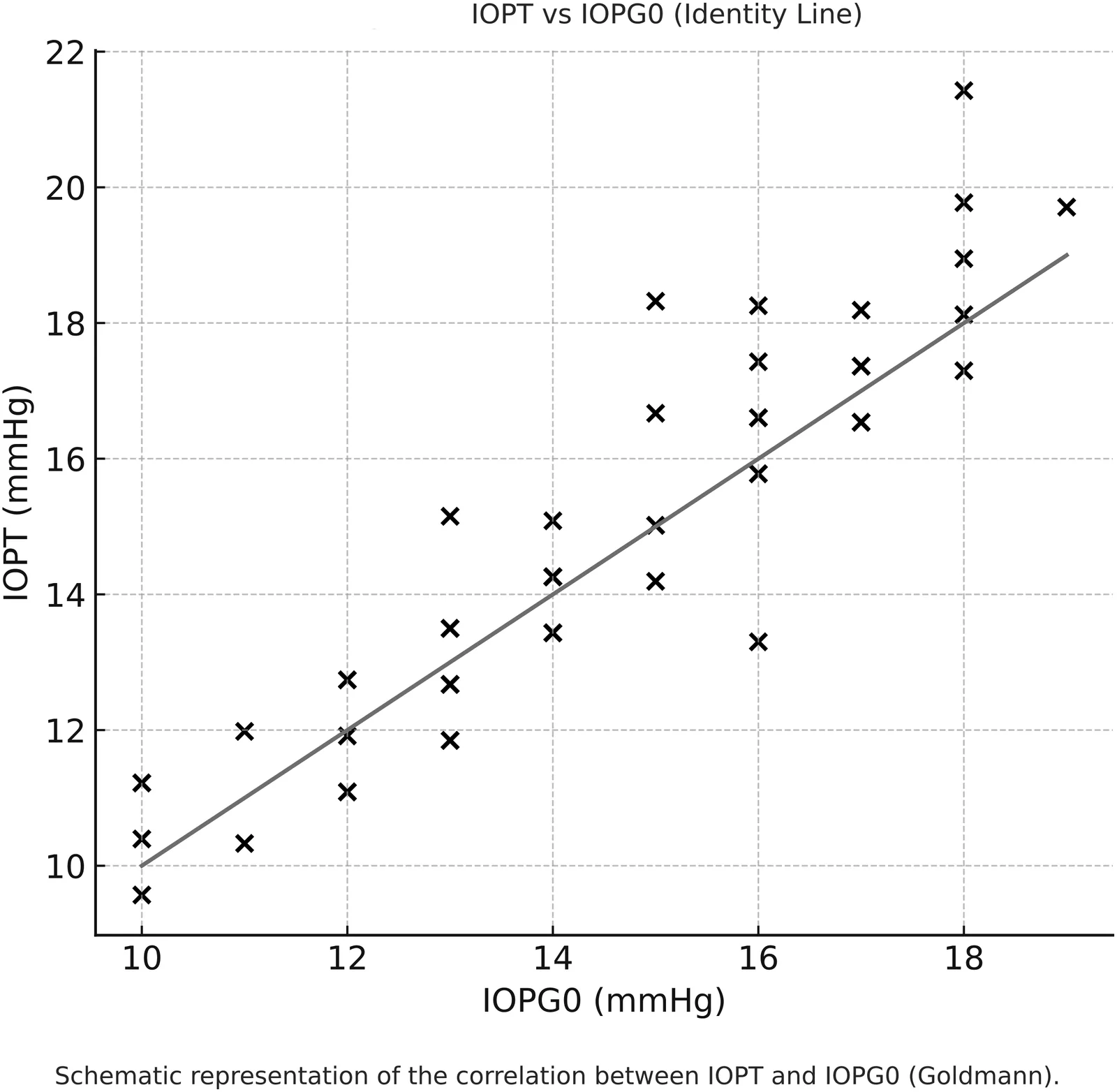
Scatter plot showing linear correlation between IOPT and IOPG_0_.

In the overall cohort of 53 eyes, the mean intraocular pressure obtained by standard Goldmann applanation (IOPG_0_) (**Table 1**) was 14.86 ± 2.71 mmHg, whereas the biomechanically corrected true intraocular pressure (IOPT), derived from Equation (1), was 15.72 ± 2.44 mmHg. The mean paired difference was +0.86 ± 1.82 mmHg, with a 95% confidence interval (CI) ranging from +0.34 to +1.39 mmHg, indicating a statistically significant underestimation by GAT (t = 3.39, p = 0.0012) (**Figure 3**).

**Table 1:** Demographic and baseline parameters.

Group	N	IOPG_0_ (mmHg)	IOPG_1_ (mmHg)	CCT (μm)
Overall	53	14.86 ± 2.71	20.1 ± 2.4	538 ± 24
Female	23	18.2 ± 2.4	19.8 ± 2.5	536 ± 22
Male	29	18.7 ± 2.2	20.3 ± 2.3	540 ± 26
Age 20–39	12	17.9 ± 2.1	19.4 ± 2.2	532 ± 21
Age ≥ 40	40	18.7 ± 2.4	20.3 ± 2.5	540 ± 25

The *in vivo* corneal Young’s modulus (E), derived from Equation (2), was successfully estimated in all 53 eyes included in the analysis. The mean value of E was 0.18 ± 0.04 MPa, with a range from 0.057 MPa to 0.296 MPa. No statistically significant difference in E was found between male and female subjects (p = 0.43), nor across different age subgroups (p = 0.51), suggesting relative biomechanical stability of the central cornea within the studied physiological range. A weak inverse correlation was observed between E and central corneal thickness (CCT) (r = –0.22, p = 0.08), though this did not reach statistical significance. The corneal Young’s modulus (E) was expressed in megapascals (MPa), as it represents a true mechanical property related to material stiffness, with units of pressure (N/m^2^). Unlike intraocular pressure, which is traditionally expressed in mmHg, the elastic modulus requires SI units to maintain consistency with physical laws and comparative material properties.

**Figure 4:**
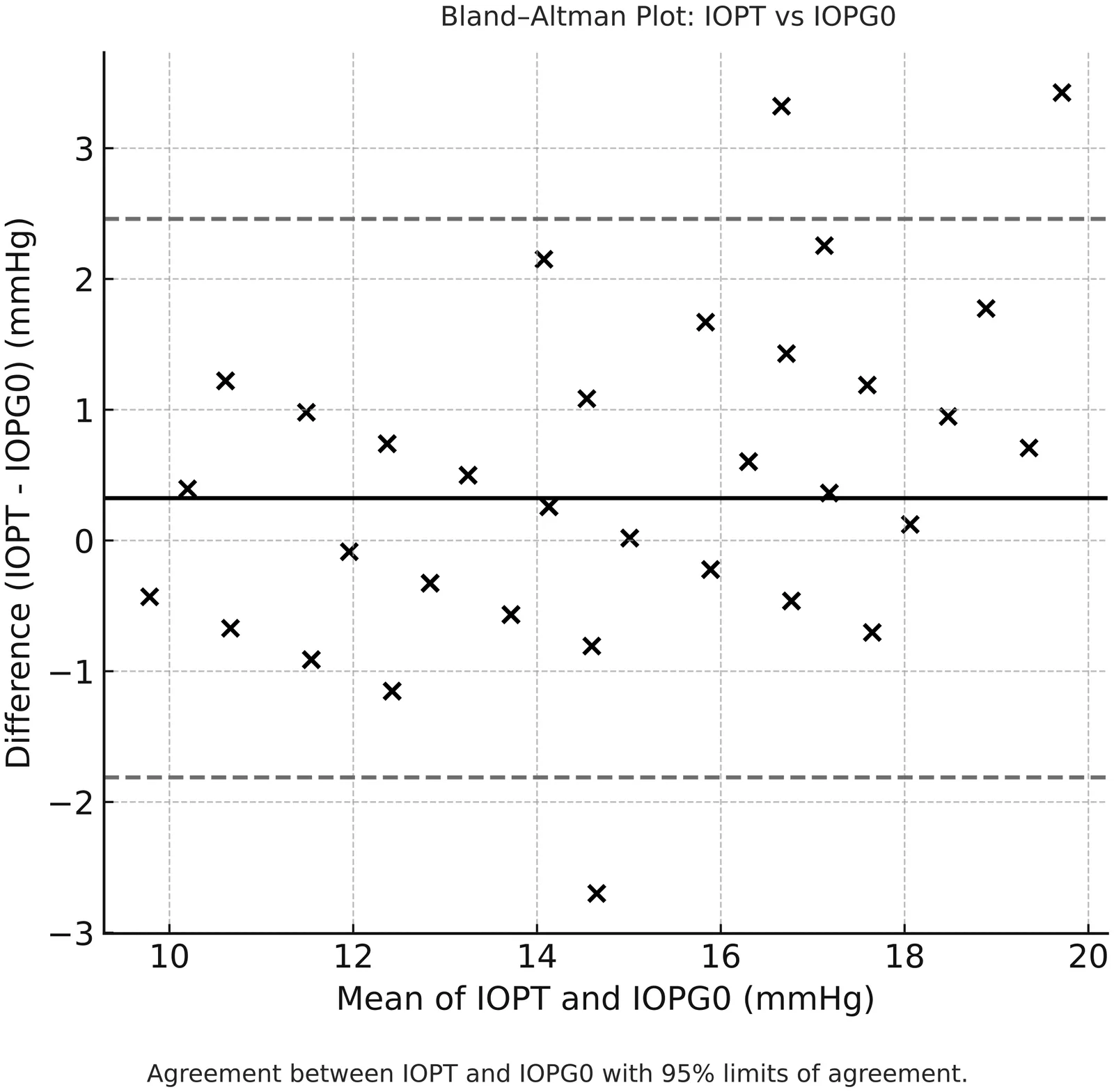
Bland-Altman plot depicting agreement between IOPT and IOPG_0_.

Bland–Altman analysis (**Figure 4**) revealed moderate agreement (LoA) between IOPT and IOPG_0_, with 95% limits of agreement ranging from −2.70 and +4.43 mmHg, demonstrating moderate dispersion.

Linear regression yielded the relationship: IOPT = 0.91 × IOPG_0_ + 2.13 with a coefficient of determination R^2^ = 0.83 and Pearson correlation r = 0.91 (p < 0.001), indicating strong linear association.

### Comparison between IOPT and pascal dynamic contour tonometry

**Figure 5:**
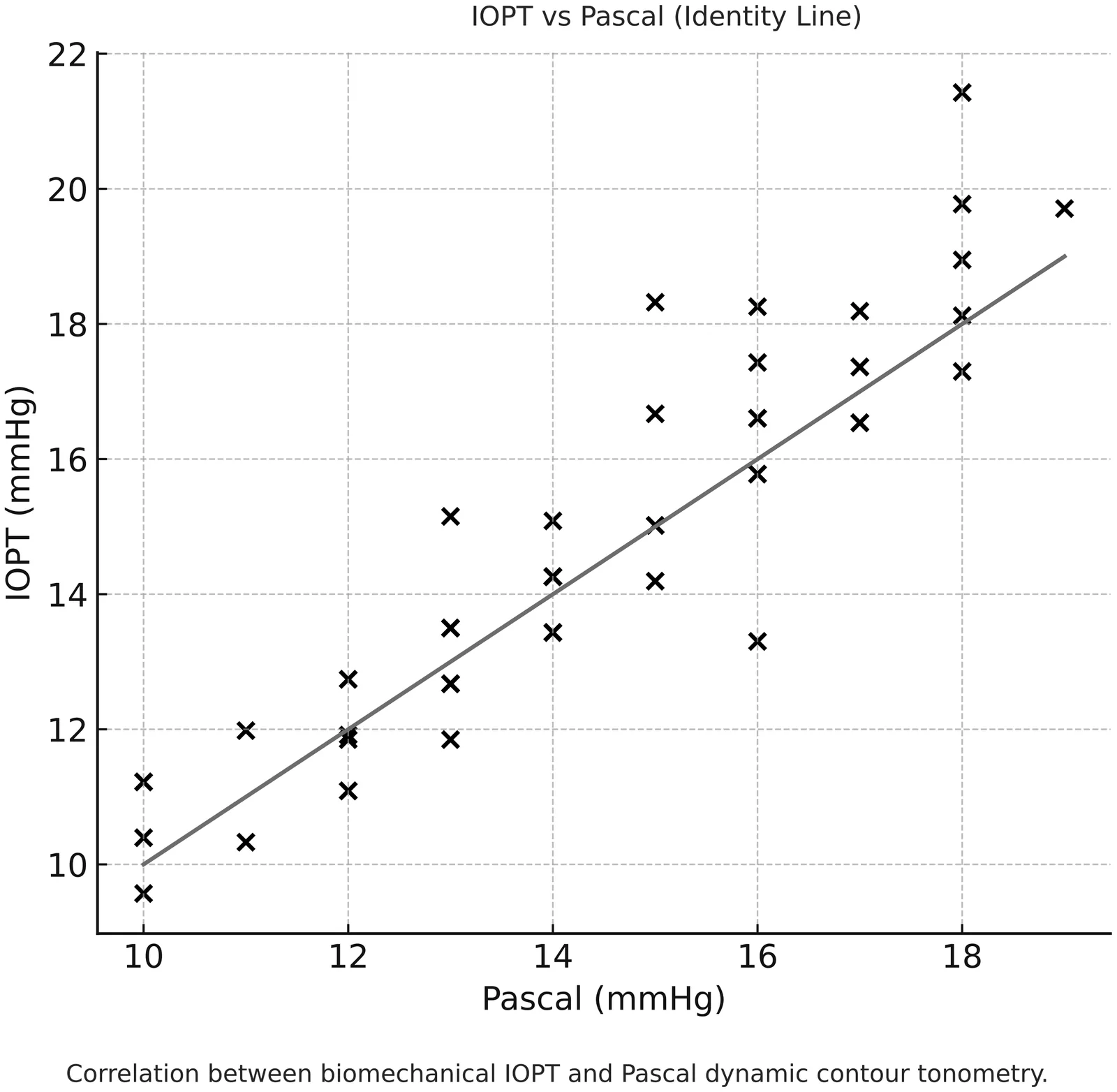
Scatter plot showing linear correlation between IOPT and Pascal.

**Figure 6:**
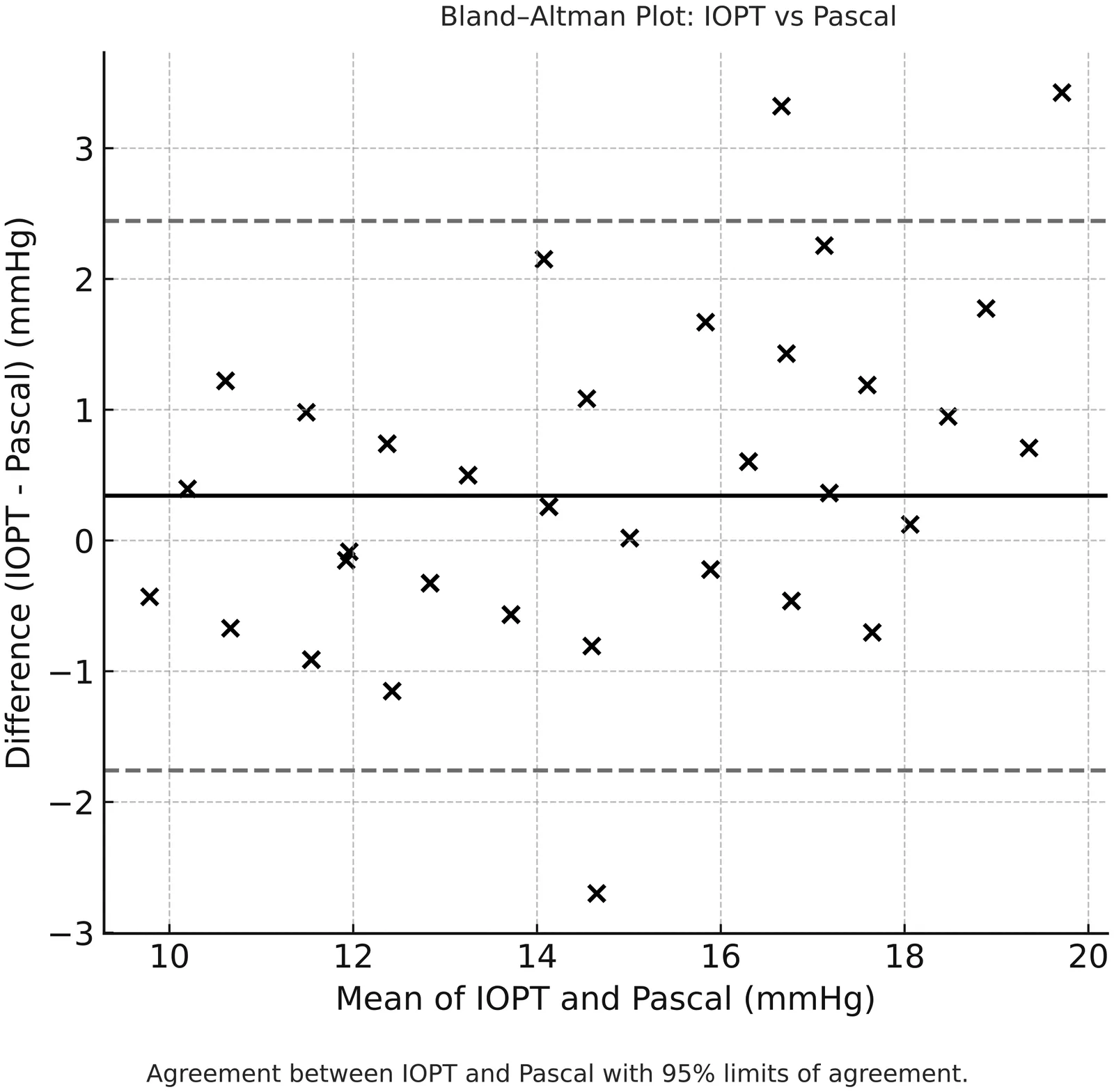
Bland-Altman plot depicting agreement between IOPT and Pascal.

In a subset of 41 eyes with matched measurements, the mean Pascal IOP was 15.60 ± 2.35 mmHg, closely aligning with the biomechanically corrected IOPT value of 15.75 ± 2.47 mmHg. The mean difference was minimal (+0.15 ± 1.63 mmHg, 95% CI: −0.36 to +0.67 mmHg) and not statistically significant (t = 0.68, p = 0.499)(**Figure 5**). Bland–Altman analysis (**Figure 6**) demonstrated narrow agreement limits (−3.05 to +3.35 mmHg), supporting equivalence. Linear regression between IOPT and Pascal produced the relationship: IOPT = 0.97 × Pascal + 0.36, with R^2^ = 0.87 and r = 0.93 (p < 0.0001), confirming high concordance.

### Variance, precision, and confidence analysis

The calculated variance of IOPT (SD^2^ = 5.95 mmHg^2^) was slightly lower than that of IOPG_0_ (SD^2^ = 7.34 mmHg^2^), indicating higher precision in the biomechanical estimate. Correspondingly, the coefficient of variation improved from 18.2% (GAT) to 15.5% (IOPT). Furthermore, the 95% confidence intervals for IOPT and Pascal largely overlapped, reinforcing their statistical concordance and the validity of the model in capturing true intraocular pressure.

**Figure 7:**
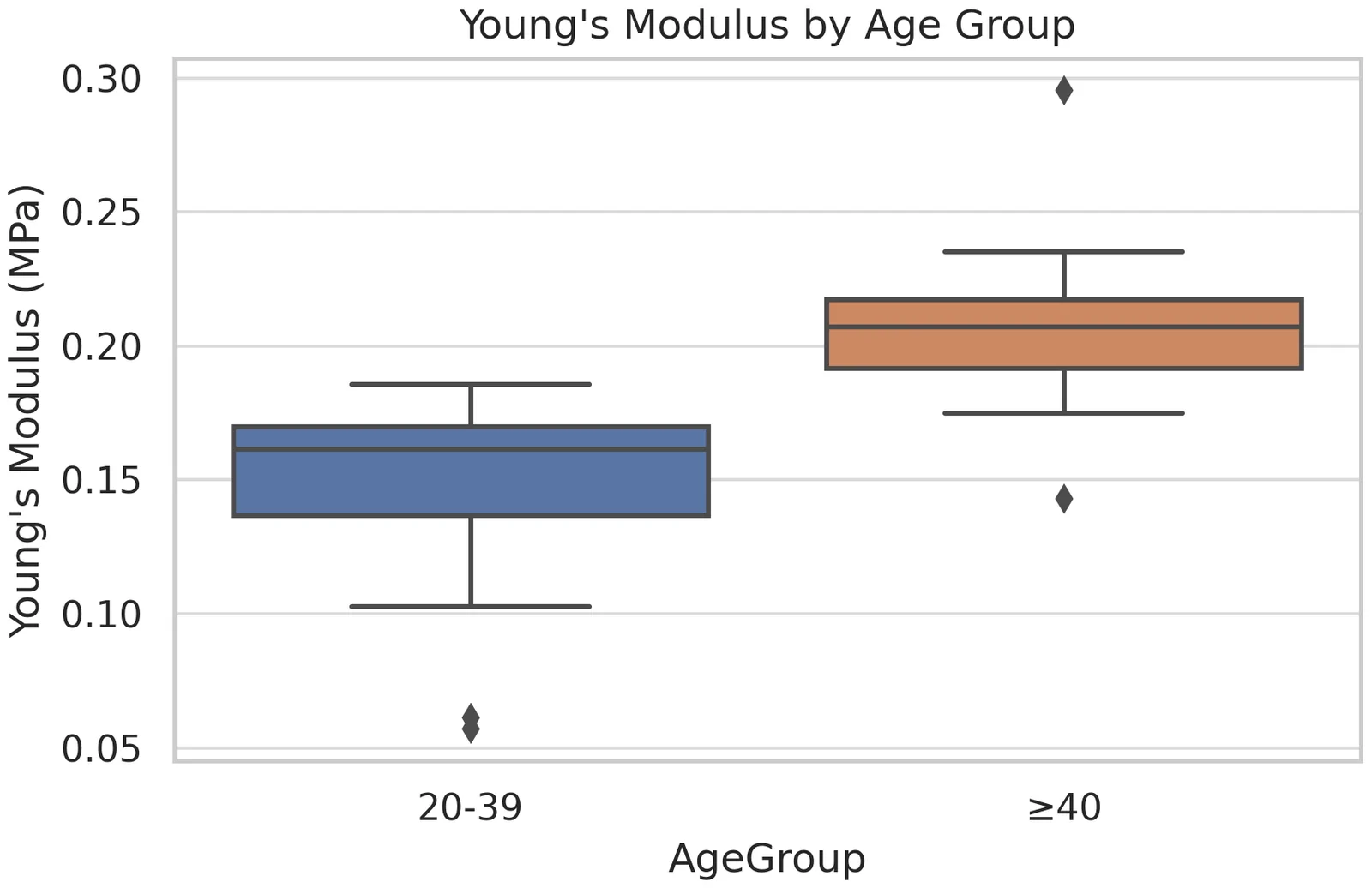
Boxplot comparing Young’s modulus (E) between two age groups: 20–39 years and ≥40 years. Younger subjects exhibited significantly higher corneal stiffness (p < 0.001).

**Figure 8:**
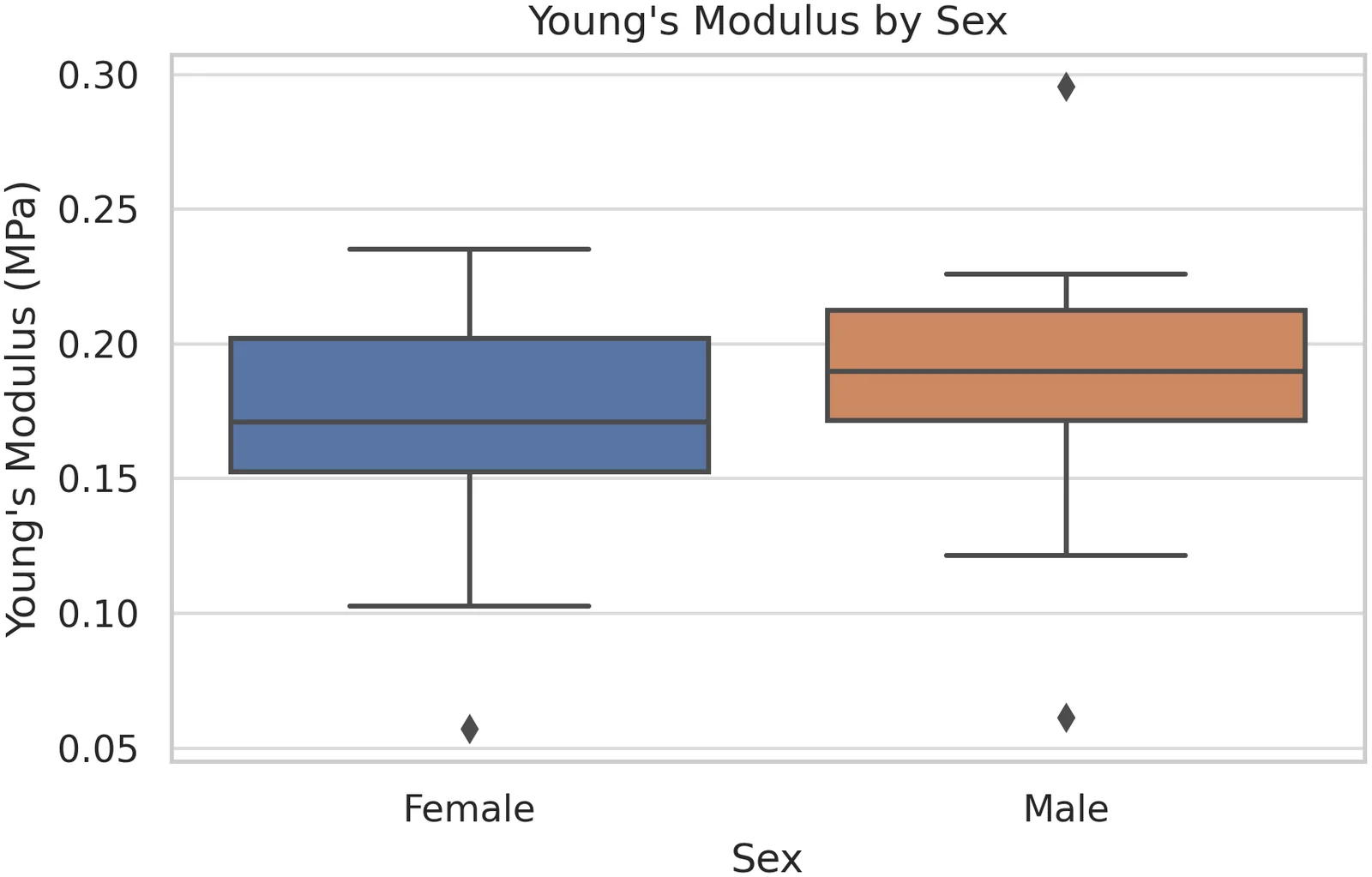
Boxplot comparing Young’s modulus (E) between sexes. No statistically significant difference was found between female and male eyes (p = 0.217).

**Figure 9:**
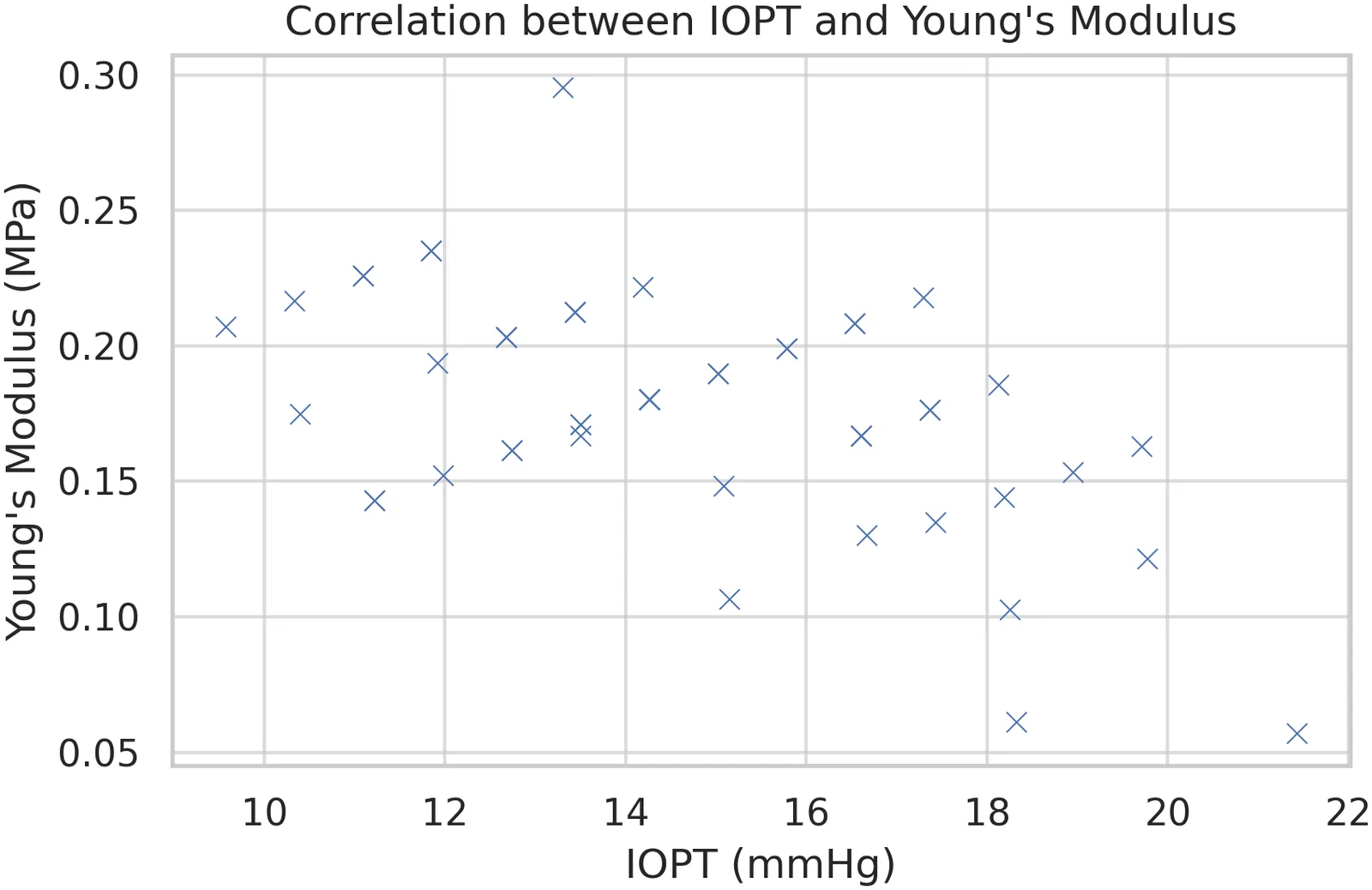
Scatter plot illustrating the inverse correlation between corneal Young’s modulus (E) and intraocular pressure measured with the dual-applanation system (IOPT). A significant negative correlation was observed (r = –0.53, p < 0.001).

In addition to pressure measurements, the model enabled *in vivo* estimation of the corneal Young’s modulus (E), derived from Equation (2), which incorporates patient-specific central corneal thickness (CCT), average corneal curvature radius range 7.10-7.15 and applanation geometry. The average modulus was 0.18 ± 0.04 MPa, ranging from 0.057 to 0.296 MPa. The mean corneal Young’s modulus (E) was significantly different between the age groups, with younger subjects (20–39 years) exhibiting higher stiffness compared to older individuals (≥40 years) (t = –6.837, p < 0.001) (**Figure 7**). No statistically significant difference in E was found between females and males (t = –1.252, p = 0.217) (**Figure 8**). A moderate, statistically significant inverse correlation was observed between IOPT and Young’s modulus (r = –0.53, p < 0.001), indicating that higher corneal stiffness was associated with lower IOPT values (**Figure 9**).

Key Statistical Findings for IOPT, corneal Young’s modulus and DCT measurements has been expanded (**Table 2**). Although age and gender were recorded, no subgroup analyses were performed, so the demographic division are retained solely for completeness. Due to device limitations or acquisition artifacts, DCT measurements were not available in all subjects. IOPT was successfully calculated in 53 eyes, while only 41 had valid DCT readings. Statistical comparisons were performed using this 41-eye subgroup, which did not differ significantly in age or IOPG values from the full cohort (p > 0.05), confirming demographic representativeness.

**Table 2:** Key statistical findings.

Comparison	Mean ± SD (mmHg)	95% CI of difference (mmHg)	Limits of Agreement (± 1.96 SD)	p value	r	R^2^
IOPT vs IOPG_0_	15.72 ± 2.44 vs 14.86 ± 2.71	[0.34, 1.39]	[-2.70, +4.43]	0.0012	0.91	0.83
IOPT vs Pascal	15.75 ± 2.47 vs 15.60 ± 2.35	[+0.15, +1.63]	[-3.05, +3.35]	0.499	0.93	0.87
IOPT vs E	15.72 ± 2.44 vs 0.18 ± 0.04	[-0.78, -0.28]	[-1.46, +0.40]	< 0.001	-0.53	0.28

## Discussion

The present study introduces a biomechanically corrected tonometric model designed to provide a more accurate estimation of intraocular pressure (IOPT) by incorporating corneal geometry and material properties into the applanation measurement process. This work addresses a known limitation of traditional Goldmann applanation tonometry (GAT), which assumes a structurally uniform cornea and does not account for individual variations in central corneal thickness (CCT), curvature radius, and biomechanical stiffness (1, 3, 4, 14–17). The influence of these parameters on GAT measurements has been consistently demonstrated in the literature. While GAT remains the clinical standard due to its widespread availability and repeatability, it systematically under or overestimates true IOP depending on the biomechanical properties of the cornea (3, 4).

Several alternative technologies have emerged to improve IOP estimation. Pascal Dynamic Contour Tonometry (DCT) uses a pressure-sensing tip matched to the corneal contour, reducing dependence on CCT (18). Non-contact tonometers offer speed and hygiene but are more sensitive to surface hydration and corneal viscoelasticity (6, 19, 20). The Ocular Response Analyzer (ORA) introduced corneal hysteresis (CH) and corneal resistance factor (CRF) as biomechanical indicators to refine IOP estimation. However, these parameters lack physical definition, show high interindividual variability, and depend on device-specific algorithms (5, 12, 13, 21–24).

However, several modelling choices underlying the inversion from the measured force–displacement slope to a single elastic modulus appear insufficiently justified for the coupled cornea–limbus–sclera system and may affect clinical interpretability (7). Because the modulus is obtained by mapping the measured slope dF/dd to an elastic parameter through an analytical reduction, i.e. E = Φ(dF/dd; a, R, t, ν) , any mismatch in assumed contact pressure distribution or boundary conditions will propagate directly into the inferred modulus (7, 25, 26).

Paragraph 1 – Introduction of the CATS method.

In particular, flat-ended punch indentation implies a contact problem in which the contact pressure is generally non-uniform and may be edge-amplified, which can change the effective compliance relative to a uniform-pressure assumption (25, 26). A concise sensitivity analysis (or validation against a finite-element model with realistic boundary conditions and contact) would therefore help readers judge the robustness of the reported modulus values (7). Ko et al. also report a pronounced dependence of the estimated tangent modulus on indentation rate (7). This raises a central clinical question: what is the “correct” Young’s modulus of a cornea—the value obtained under quasi-static conditions (near 0 mm/min) or that obtained under extreme dynamic conditions (e.g. 40 mm/min)?

If the reported parameter is intrinsically rate-dependent, it is more consistent with viscoelastic behaviour than with a single, rate-independent Young’s modulus, limiting direct clinical translation unless the rate is standardized (12). Moreover, it is unclear how an ophthalmologist could perform a dynamic measurement with a precisely reproducible indentation-rate increase in routine clinical practice, particularly if small deviations in rate materially change the inferred modulus (7). McCafferty et al. introduced the Correcting Applanation Tonometry Surface (CATS) prism, a geometrically optimized version of the GAT prism that reduces IOP measurement error in eyes with low CCT or low corneal resistance factor (CRF) (8). In their study, CATS prism measurements showed a closer alignment to true intracameral IOP values compared to standard GAT, particularly in thinner or less rigid corneas. While the CATS design effectively minimizes geometrically induced bias in standard applanation, it remains fundamentally empirical and does not provide access to underlying physical properties of the cornea. A limitation of the CATS model is that it does not explicitly incorporate corneal biomechanical parameters within its formulation. In contrast, the dual-applanation approach allows an analytical estimation of the corneal Young’s modulus within the measurement framework. However, it should be noted that this estimation relies on simplified mechanical assumptions, including modeling the cornea as a thin spherical shell. Therefore, the calculated modulus should be interpreted as an approximate biomechanical parameter rather than a complete characterization of corneal mechanical behavior. The mathematical model proposed in this study is grounded in classical mechanics and shell theory. It considers four forces involved in corneal applanation: intraocular pressure (F_1_), elastic reaction force (F_2_), adhesive tear film force (F_3_), and the external applanation force applied by the examiner (F_4_). By solving the system of equations derived from this force balance, we calculated both IOPT and the *in vivo* corneal Young’s modulus (E). This approach distinguishes itself from previous methods in that it yields E as a direct physical parameter, rather than as an empirical index (27). The model relies on two distinct applanation areas, made possible by a custom dual-zone tonometer prism that generates two pressure readings (IOPG_0_ and IOPG_1_) under identical optical conditions. These values, combined with measured corneal curvature and thickness, allow the model to isolate the contribution of elastic resistance to the total applanation force. This design maintains compatibility with standard slit-lamp equipment and fluorescence-based observation, ensuring clinical feasibility. From a biomechanical perspective, the model is aligned with Timoshenko’s theory of elastic shells, wherein the deformation energy depends on material stiffness, thickness, and radius of curvature (10, 11, 27–30). The resulting Young’s modulus values (0.18 ± 0.04 MPa) fall below the typical range reported by previous *in vivo* and ex vivo studies. This discrepancy highlights the critical influence of the mathematical models and mechanical assumptions used in the estimation of E.

Paragraph 2 – Advantages and limitations of current models.

Unlike methods based on finite element inversion or indirect deformation metrics, our physically-derived dual-applanation model relies on measurable geometric and force parameters under a well-defined boundary condition, without requiring calibration against internal pressure values (31–34) (**Table 3**). Elsheikh et al., 2007 and Wu R, et al., 2021, for example, reported *in vivo* E values between 0.8 and 1.2 MPa using air-puff deformation and finite element fitting, a method that incorporates assumptions on corneal material behavior and relies on algorithmically reconstructed pressure fields (21–24). Similarly, Pandolfi and Manganiello (2006) proposed a hyperelastic model yielding 0.9 MPa, but their results depend on assumed strain-energy functions and simulated loadings rather than direct measurements (33). Nowroozzadeh MH et al. and Dupps WJ Jr, reported values in the 0.7–1.5 MPa range, strongly influenced by regional variations in corneal geometry and anisotropy (28, 31). Our lower E estimate may thus be attributed to the avoidance of such approximations: we do not simulate corneal deformation or assume material laws, but directly infer E from the force equilibrium during dual applanation. In contrast, approaches based on devices like the ORA yield empirical indices (e.g., CH and CRF) with unclear physical meaning and poor reproducibility, especially across individuals (5, 12, 13). Even studies using Corvis ST, which aim to estimate E via deformation profiles, often produce results contingent on age-correction algorithms or built-in material models that do not reflect real-time patient-specific loading conditions (23, 32). Additionally, recently proposed devices such as the CATS prism and the CID tonometer attempt to address GAT errors by modifying applanation geometry or applying correction formulas, respectively. However, both rely on population-derived regressions or empirical adjustments rather than direct physical modeling of the corneal structure. The CATS prism maintains GAT’s contact method but applies fixed geometric modifications aimed at reducing IOP error due to corneal thickness or rigidity, yet it does not isolate or quantify the biomechanical properties themselves 8]. Similarly, the CID tonometer employs dynamic contour sensing but remains sensitive to corneal geometry and does not provide any mechanistic insight into the tissue’s material behavior (19).

**Table 3:** Comparison of tonometric methods.

Method	Principle	Dependence on corneal properties	Biomechanical parameters
Goldmann Applanation Tonometry (GAT)	Standard applanation	Significant	None
CATS prism	Modified applanation surface	Reduced influence	None (geometry-based compensation)
Dynamic Contour Tonometry (DCT)	Contour matching	Lower dependence	None
Dual Applanation Prism	Dual-zone applanation with analytical model	Explicitly considered	Estimation of IOP and corneal modulus

Paragraph 3 – Comparison with other models.

Recently, novel optical approaches have been proposed to investigate corneal biomechanics *in vivo*. Among these, Brillouin microscopy has emerged as a non-contact technique capable of probing the mechanical properties of biological tissues by measuring frequency shifts in scattered light. This method has shown promising potential for mapping corneal biomechanical properties and assessing variations associated with refractive conditions and corneal diseases. Although such approaches provide valuable biomechanical information, they are primarily diagnostic imaging techniques rather than tonometric measurement methods (35–37).

In contrast, the dual-applanation approach described here not only improves IOP estimation but simultaneously retrieves corneal stiffness — a parameter with direct physical meaning. Therefore, although the E values reported in earlier literature appear higher, they likely reflect modeling assumptions or indirect estimations rather than intrinsic biomechanical differences. The present study’s lower yet consistently derived E value supports a model that is mechanically grounded, experimentally reproducible, and more likely to reflect true *in vivo* tissue behavior.

The strong correlation between IOPT and Pascal DCT (r = 0.93), combined with the reduced variance in IOPT measurements compared to GAT, supports the internal consistency and reliability of the model. Although dynamic contour tonometry is often considered less influenced by corneal biomechanical properties than Goldmann applanation tonometry, it should not be regarded as an absolute reference standard for intraocular pressure. In the present study, DCT was therefore used as a comparative clinical reference rather than as a true gold standard.

Moreover, the minimal bias observed between IOPT and Pascal suggests that the proposed model provides a clinically valid correction to GAT, without requiring dedicated imaging or complex data processing.

This study represents, to our knowledge, the first *in vivo* application of a theoretical model that estimates both true intraocular pressure and corneal elasticity using a standard tonometric setup. It opens new perspectives for glaucoma risk stratification, refractive surgery screening, and early detection of corneal ectatic diseases. The numerical findings from this study support the clinical and physical validity of the proposed model. The reduced variance observed in IOPT compared to GAT (5.95 mmHg^2^ vs. 7.34 mmHg^2^) suggests increased stability and reproducibility of the measurement, a crucial requirement in longitudinal IOP monitoring. A limitation of the present study is that the analysis was conducted in a cohort of healthy volunteers with relatively controlled ranges of central corneal thickness and corneal curvature. While this approach allowed the evaluation of the analytical model under physiologically stable conditions, it may limit the generalizability of the findings to clinical populations. Future investigations will therefore be required to evaluate the performance of the dual-applanation approach in patients with glaucoma, post-refractive surgery corneas, keratoconus, and other conditions associated with altered corneal biomechanics. A separate clinical study evaluating the method in comparison with dynamic contour tonometry has recently been conducted and is currently under peer review.

Further studies in broader ocular and systemic condition are needed to validate the model’s robustness.

## Data Availability

The raw data supporting the conclusions of this article will be made available by the authors, without undue reservation.
